# Effect of *Daucus carota* L. extract on spermiogram factors in men with idiopathic infertility: A double‐blinded randomized clinical trial

**DOI:** 10.22038/AJP.2024.25008

**Published:** 2025

**Authors:** Abolhasan Mousavi Khorshidi, Ayesheh Enayati, Nasser Behnampour, Emadoddin Rezaei, Fatemeh Kolangi

**Affiliations:** 1 *Clinical Research Development Unit [CRDU]* *,* * Sayad Shirazi Hospital, Department of Persian Medicine, School of* *Persian Medicine, Golestan University of Medical Sciences, Gorgan, Iran*; 2 *Ischemic Disorders Research Center, Golestan University of Medical Sciences, Gorgan, Iran*; 3 *Biostatistics Department, Faculty of Health, Golestan University of Medical Sciences, Gorgan, Iran*; 4 *Counseling and Reproductive Health Research Center, Department of Persian Medicine, School of Persian Medicine, Golestan University of Medical Sciences, Gorgan, Iran*

**Keywords:** Male infertility, Persian Medicine, Daucus carota L., Clomiphene, Lifestyle

## Abstract

**Objective:**

Male infertility is a common issue that affects people worldwide and presents challenges in terms of treatment. In recent times, there has been significant interest in the use of herbal remedies as a potential solution for male infertility. In this study, we aimed to assess and compare the effects of clomiphene, carrot seed, and education based on traditional Persian medicine on the sperm parameters in idiopathic male infertility.

**Materials and Methods:**

Sixty male patients experiencing infertility were randomly divided into four groups: Clomiphene, Clomiphene + Carrot, Clomiphene + Lifestyle modification, and Clomiphene + Carrot + Lifestyle modification. In the herbal group, patients received four capsules of carrot seeds, for a total of 2 g daily for 90 days. Meanwhile, subjects in the Clomiphene group were administered one tablet of clomiphene, containing 50 mg, per day for a duration of 90 days. Sperm parameters were analyzed at the beginning and end of the study.

**Results:**

After the 90-day intervention, the groups that received clomiphene combined with carrot and lifestyle interventions showed significant improvements in various sperm parameters. These improvements were statistically significant compared to the control group.

**Conclusion:**

The combination of carrot seeds along with clomiphene and education based on traditional Persian medicine was found to improve sperm parameters in cases of idiopathic male infertility without any adverse effects.

## Introduction

Various approaches have been developed for addressing male factor infertility (Alfaro Gómez et al., 2023). However, assisted reproductive technology (ART) procedures are costly, and invasive, and have relatively low success rates. As a result, there is a growing preference among both patients and clinicians for pharmacological treatments as a more viable option (Nasimi Doost Azgomi et al., 2018).

Clomiphene citrate (Clomiphene), also known as Clomid, belongs to a class of medications called selective estrogen receptor modulators (SERMs). It functions by binding to estrogen receptors in the hypothalamus and pituitary gland, which leads to the stimulation of the hypothalamic-pituitary-gonadal (HPG) axis. This stimulation results in the release of gonadotropins, which in turn, enhance testosterone production (Surampudi et al., 2014; Huijben et al., 2023a). The U.S. Food and Drug Administration (FDA) has not officially approved clomiphene therapy for male infertility and hypogonadism. Nevertheless, it has been prescribed off-label for these conditions (Thaker et al., 2020; Campbell et al., 2021). According to a survey conducted by the American Urological Association in 2012, and later revised in 2020, a significant percentage of male fertility fellowship-trained urologists (78% and 93% respectively) reported using clomiphene in addition to other empirical therapies and surgical interventions for the treatment of infertile males (Thaker et al., 2020; Campbell et al., 2021).

Panner Selvam et al. reported that clomiphene treatment resulted in improved testosterone levels in men with low sperm concentration (Panner Selvam et al., 2023). These findings align with prior research indicating that clomiphene treatment leads to enhancements in semen parameters (Huijben et al., 2023a; Huijben et al., 2023b). However, it is important to note that the use of clomiphene can also result in side effects such as headache, dizziness, flushing, acne, irritability, pectoral muscle tenderness, stomach upset, bloating, or abdominal/pelvic fullness (Guo et al., 2020). Given these factors, there is an urgent need to explore and implement alternative strategies for addressing male infertility (Ahmad et al., 2010). 

Complementary and alternative medicine, particularly herbal medicine, has gained significant attention due to its high efficacy, low rate of adverse effects, and social acceptance (Nasimi Doost Azgomi et al., 2018). Persian Medicine has addressed various issues related to sexual function, including anatomical abnormalities of the male reproductive system, erectile dysfunction, abnormalities in sperm parameters, and decreased libido (Tahvilzadeh et al., 2016). Persian Medicine researchers have proposed specific treatment approaches, with a focus on the use of medicinal plants which are known for their positive effects on organs such as the brain, heart, and testes, as well as their favorable side effect profiles (Nejatbakhsh et al., 2016).

In Persian Medicine, a range of medicinal plants, including *Daucus carota*, are employed in the treatment of male sexual disorders. *Daucus carota* is described in Persian Medicine as having a warm and moist nature, while its seeds possess a warm and dry temperament. *Daucus carota *subsp*. carota,* (Carrot) also referred to as Persian carrot, *Zardak*, *Gazar*, and *Jazar*, belongs to the Apiaceae family (Kolangi and MousaviKhorshidi, 2023). According to Persian Medicine sources, the seed of Carrot, known as "*doghu*," is considered the most potent part of the plant. It is believed that the seed of carrot, particularly the garden variety, has a positive impact on sexual function (Kolangi and MousaviKhorshidi, 2023). Carrot contains terpene compounds such as cedarone, azarone, and geranyl acetate, which are present in its volatile oil as oxygenated sesquiterpenes and monoterpenes. Additionally, it contains polyphenol compounds such as alkaloid compounds, coumarins, flavonoids, and various steroids, all of which possess antioxidant properties (Tijjani et al., 2019). Recent studies have highlighted the efficacy of polyphenolic compounds present in carrot, including hydroxycinnamic acid, flavonoids, and anthocyanins, in the treatment of infertility (Ganguly et al., 2020). The antioxidant activity of these compounds, have the potential to enhance sperm function and protect them from oxidative stress. All kinds of oxidative stress have been known to decrease sperm count, motility, and normal morphology in men (Alahmar, 2018). Animal studies have demonstrated that the extract of carrot seeds can significantly increase levels of luteinizing hormone (LH) and testosterone (Nouri et al., 2009; Abouzaripour et al., 2024).

However, there have been no randomized clinical trials conducted to evaluate the effects of carrot on the mentioned parameters or to compare its effects with standard medications, such as clomiphene. Therefore, this study aimed to compare the efficacies of *D. carota* subsp. *carota* and clomiphene citrate on sperm parameters in idiopathic male infertility.

## Materials and Methods

### Trial design

This randomized double-blind controlled clinical trial was approved by the Committee of Medical ethics, Golestan University of Medical Science (approval code: IR.GOUMS.REC.1400.319), and was registered by the Iranian Registry of Clinical Trials (registration ID: IRCT20210911052430N1). It was conducted in Nahal Gorgan Infertility Treatment Clinic of Golestan University of Medical Sciences, Gorgan, Iran between December 2021 and September 2023. All of the data about the participants were kept confidential by the researchers. All stages of the study were based on the principles of Helsinki convention. Informed consent was received from all participants.

### Study protocol

The study population was comprised of male patients who were experiencing infertility and were between the ages of 20 and 50 years. Infertility was defined as the inability to achieve conception after engaging in unprotected sexual intercourse for a duration of 1 year or more. In the study, written informed consent was obtained from all the patients. The inclusion criteria were as follows: (1) Idiopathic abnormal spermiogram defined as: Sperm count <15 million/ml or <39 million/specimen (according to World Health Organization criteria) (Cooper et al., 2010), sperm percentage with normal motility <40% (progressive motility <32%) or sperm percentage with normal morphology <30% (<4% based on strict criteria) and semen volume <1.5 ml; (2) Body Mass Index (BMI) less than 30 kg/m^2^; (3) Not having a history of hyperprolactinemia or androgenic diseases, as well as a total sperm count below 5 million; (4) No uncontrolled systemic illnesses including diabetes mellitus, thyroid disorders, uncontrolled hypertension and cerebral hemorrhage, liver and gallbladder cancers, or renal failure; (5) Non-smoker and not addicted to opiates or alcohol; (6) No urogenital infection or anatomical abnormality of this, including varicocele; (7) No prior history of testicular trauma; (8) Absence of unilateral testicular atrophy; (9) No prior history of pelvic area surgery; or (10) No prior history of chemotherapy or treatment with anticoagulants, testosterone, corticosteroids, or anti-androgen medications within the 8 weeks preceding the study.

Patients who used less than 80% of the prescribed medications, patients who experienced severe adverse effects related to the medications used in the study, or patients who were concurrently using other medications that could potentially interfere with the study outcomes, were excluded from the study.

The samples were not under infertility treatment for at least three months before the intervention, and after being selected for the study, they were instructed not to use any other treatment method. They were also trained to inform the researcher if they want to use new drugs and supplements.

All samples were visited by a urologist before entering the study. Their wives were examined in terms of fertility at the Nahal Infertility Center of Sayad Shirazi Hospital, Gorgan, Iran, and they were included in the study after ruling out female infertility and according to WHO 2010 spermiogram criteria (Cooper et al., 2010). In the Nahal infertility center, all couples are under the supervision of a gynecologist.

### Study outcomes

The primary outcome of this study was the impact of the medications on improving sperm parameters, including sperm morphology, sperm motility, sperm count, semen volume, and total motile sperm count (TMSC) in infertile males after a 90-day treatment period. The study aimed to compare these improvements between the different groups under investigation. As a secondary outcome, the study also aimed to evaluate the occurrence of major adverse effects in the studied groups (Bjorndahl and Brown, 2022).

### Preparations of carrot seed

The seeds of carrot were purchased from a herbal medicine market in Tehran, Iran. These seeds were then confirmed and verified by a certified botanist and assigned a registration herbarium code (popular marketing plant-PMP) at the Faculty of Pharmacy (Voucher number: PMP-3657), Tehran University of Medical Sciences, Tehran, Iran. The extraction and sample preparation followed a method proposed by Abubakar and Haque (Abubakar and Haque, 2020), with some modifications. The carrot seeds were first washed and dried. To obtain a hydroalcoholic extract, a specific amount of the herbal material was macerated in 70% ethanol for four days at room temperature. Methylparaben and propylparaben were dissolved using propylene glycol, stirred until became homogeneous, and then added into the extract. Then, the extract was filtered and dried via spray-drying.

### HPLC profiling of the carrot seed extract

To standardize the plant material, the HPLC (High-Performance Liquid Chromatography) method was employed to determine the content of quercetin, which is the main bioactive constituent of carrot seed. Quercetin standard was purchased from Sigma Aldrich (#Q4951). The hydroalcoholic extract of the plant material was standardized using the HPLC method with the following specifications: C18 (250 mm × 4.6 mm) column, UV detector wavelength of 260 nm, and the mobile phase was composed of solvent A: water: acetic acid: methanol (10:2:88) and solvent B: water: acetic acid: methanol (90:2:8) at a flow rate of 1.0 ml/min. By employing this HPLC method, the hydroalcoholic extract of the plant material was standardized, ensuring the presence and quantification of quercetin as the main bioactive compound.

### Microbial tests

The microbial limit tests were performed in accordance with the British pharmacopeia for herbal preparations (Commission, 2014), in the Food and Drug Administration Laboratory in Gorgan University of Medical Sciences.

## Methods

In this study, the patients were evaluated by a urologist. Following a physical examination and necessary laboratory assessments, a total of 60 patients were considered eligible for inclusion and were selected using convenience sampling. Fixed-size block randomization was employed to allocate the subjects into four treatment groups, with each group consisting of 15 patients (n=15). The patients were distributed among four groups in blocks of 8, the blocks consisted of 4 letters with 2 repetitions in each block, which were implemented as 7 blocks of 8 and one block of 4. The randomization sequence for patient allocation was generated using the Randlist version 11 software package. 

All steps involved in the preparation of the drug, including extraction and pill-making, were carried out at the Niak Pharmaceutical Company (Golestan province, Iran). Clomiphene citrate (50 mg) tablets were purchased from Iran Hormone Company, Tehran, Iran. The capsules and tablets were then placed into opaque packets and assigned with numbers to ensure blinding. To prevent any potential bias related to smell, both the herbal medication capsules and clomiphene tablets were stored in the same place. The herbal capsules and Clomiphene were packaged together, with identification codes matching those of the questionnaires. Only the pharmacist had knowledge of the contents of each package, ensuring that both the researchers and patients remained blind to the drug allocation. The study methods were thoroughly explained to all participants, and written informed consent was obtained from each of them.

### Protocol

In this study, 60 eligible subjects were randomly allocated to four groups (n = 15 each). The groups were defined as follows: 

(i) Clomiphene group: Participants in this group received treatment with Clomiphene (50 mg tablet once daily) for a duration of 90 days.

 (ii) Clomiphene + carrot seed group: Participants in this group received treatment with both clomiphene (50 mg tablet once daily) and carrot seed capsule (a total of 2-gram carrot seed capsule daily :2 capsules/12 hr (2 times daily)) for a duration of 90 days.

 (iii) Clomiphene + traditional Persian medicine (TPM)-based sexual health training group: Participants in this group received treatment with clomiphene (50 mg tablet once daily) along with traditional Persian medicine-based sexual health training for a duration of 90 days.

(iv) Clomiphene + carrot seed + TPM-based sexual health training group: Participants in this group received treatment with clomiphene (50 mg tablet once daily), carrot seed capsule (a total of 2-gram carrot seed capsule daily: 2 capsules/12 hr (2 times daily)), and traditional Persian medicine-based sexual health training for a duration of 90 days.

 The allocation of participants to these groups was done randomly to ensure unbiased distribution of subjects across the different treatment groups.

### Collection of sperm samples and analysis

After refraining from sexual intercourse for 72 hr, semen samples were obtained from the subjects and collected in sterile plastic boxes at the beginning and after 90 days of treatment. The collected semen samples were stored at a temperature of 37°C and analyzed within one hour of being collected. The semen parameters such as the volume of semen, sperm count, motility, and morphology, were assessed based on the criteria established by the World Health Organization in 2010 (Alshahrani et al., 2018). The sperm count, the proportion of spermatozoa with normal morphology, and the percentage of motile sperm were objectively determined through examination under a microscope (Oliveira et al., 2011). Two skilled technicians, who were unaware of the study details, conducted the semen analysis.

### Collection of possible adverse effects of the medications

To gather information on potential negative effects of the treatment, a specific form was utilized for this purpose. Furthermore, the patients underwent regular physical examinations conducted by the specialist to assess any side effects. A phone call was made to the couple once a week. In every phone call, questions were asked about how to take medicines, therapeutic effects and any side effects. For this purpose, the checklist of drug side effects and the checklist of sexual health opinions of Persian medicine were used. Medicines were delivered to patients in person once a month and follow-up was done during delivery. The information obtained in all calls and in-person follow-ups was recorded in the patients' files. At the end of 3 months of treatment, the patients were visited again by the urologist after the sperm test.

### Training intervention based on traditional Persian medicine (lifestyle modifications)

The health maintenance recommendations derived from traditional Persian medicine books encompassed various fundamental principles pertaining to sexual function (Sina, 2013). The participants were advised to Preparation of the wife before intercourse (stimulating and preparing the wife at the beginning of intercourse in order to make it more enjoyable and ejaculation at the same time) (Aqili khorasani, 1385). Having sex only when there is a real desire to have intercourse, the person feels healthy and strong in the body, and is not tired, anxious or sick and erection has occurred without external stimulation. It should be done after digesting the food in the stomach, which is on average about two to three hours after eating and in mild weather, moderate body temperature and in the early hours of the night (Ibn-e-sina, 2005). Appropriate frequency of intercourse (to the extent that a person feels light and cheerful after intercourse, on average once every three days) (Aqili khorasani, 1385). Compliance with the necessary conditions after intercourse included avoiding cold weather, not drinking cold water, not washing the body or genital area with cold water after intercourse, emptying the bladder, eating a few sweet and fatty bites and rest for recuperation (Maohamaad, 1388). Enhancement of potency includes: warming up the back of the kidneys, rubbing fragrant oils on the body while sleeping, gentle leg and sole massage while sleeping and removing pubic hair (Arzani, 1391). Adjusting sleep and following a daily exercise pattern. Minimizing stressful and frightening factors. Proximity position, if possible, in such a way that the woman is lying on her back and inclined to a sitting position (Arzani, 2008). The guidelines for sexual health life style modifications of Iranian medicine were compiled according to the sources of Iranian medicine and a checklist with 26 questions was prepared based on it. Samples participating in the study were followed up with this checklist on a monthly basis during the delivery of the medicine and received a score of 0 to 26 in terms of compliance with these life style modifications. Then, the degree of adherence to these opinions and the spermiogram parameters of the samples, were modeled.

### Statistical analysis

Quantitative data are presented as mean (±standard deviation) and categorical data are reported as frequency (percentage). The normality of the data was assessed using the Shapiro - Wilk test. The Chi-square test and Fisher's exact test were used for categorical data and ANOVA and Kruskal-Wallis was used for others data. For making comparison between before and after treatment data of each group in each of the variables, based on the presence or absence of data normality, paired t test or Wilcoxon signed-rank test was used respectively to comparison of variable means before and after intervention ​​in each group.

Analysis of covariance (ANCOVA) and Generalized Estimating Equation (GEE) were used to compare the means after the intervention by adjusting the difference in the baseline value and for post hoc multiple comparisons Bonferroni's Correction test was used.

## Results

### HPLC quantitation of carrot seeds extract

**Figure 1:**
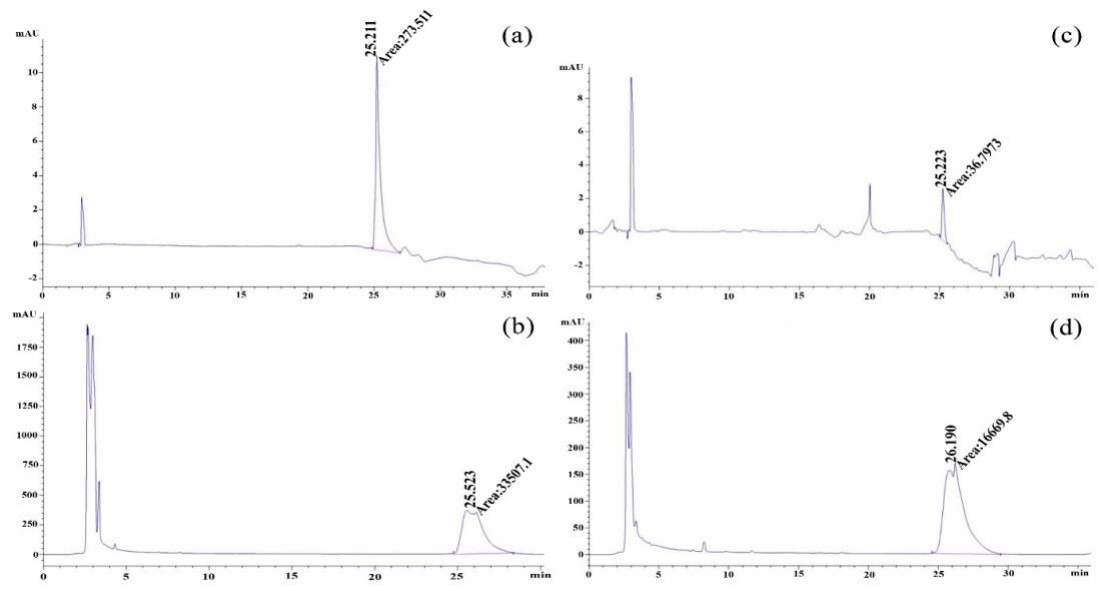
The HPLC chromatogram of quercetin standard, and the extract of Carrot seeds, at 270 nm (a and b), and 360 nm (c and d), respectively.

The obtained HPLC chromatogram of standard quercetin (Figure 1) showed a retention time at 25.21 min, and 25.22 min at 270 nm, and 360 nm, respectively. This analysis showed that the amount of quercetin was 8.2 mg/g of carroct seeds dry extract.

### Microbiological analysis of carrot seeds extract

The microbiological activity detected in the extract of carrot seeds was found to be within the acceptable limits set by the European Union (EU) for herbal medicine. This indicates that the extract complied with EU standards regarding microbial safety and was considered safe for use (Table 1).

### General study characteristics

**Figure 2:**
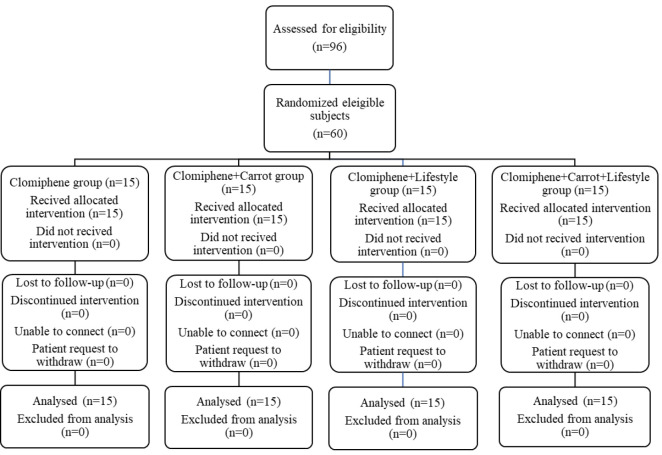
CONSORT flow diagram of participants

Out of the initial 96 participants assessed for eligibility, 36 individuals were excluded from the study. The trial ultimately 60 infertile males were enrolled and randomly assigned to four groups: Clomiphene, Clomiphene + Carrot, Clomiphene + Lifestyle modification, and Clomiphene + Carrot + Lifestyle modification. Each group consisted of 15 participants, as illustrated in Figure 2.

There were no significant differences in the baseline characteristics of the demographic characteristics between the four groups (Table 2). None of the participants in any of the four groups had a history of drug use, childhood illnesses, specific occupations, urologic surgeries, or family history of diseases. These variables were assessed, and it was determined that they did not have any discernible impact on the outcome of the study.

### Primary study outcomes

After 90 days of intervention, the total sperm count, sperm concentration, total Sperm motility, and TMSC parameters in two groups Clomiphene + Carrot and Clomiphene + Carrot + Lifestyle modification increased significantly as compared to the beginning of the study (p<0.05). In addition, a significant increase in terms of progressive motility (P-Value=0.002), and normal morphology (P-Value=0.01) was detected with Clomiphene + Carrot + Lifestyle therapy after intervention as compared to ?before. No statistically significant difference among the four groups was observed with regard to semen volume (Table 3).

Our findings showed a significant difference in some sperm parameters between patients in the clomiphene + carrot + lifestyle modification group and the clomiphene group (as a control group) (Table 4). We found that simultaneous use of carrot and lifestyle modification significantly increased the mean total sperm count (P-Value=0.01), sperm concentration (P-Value=0.007) and TMSC (P-Value=0.002) compared to the control group.

### Secondary study outcomes

Both the participants and clinicians involved in the study reported no significant adverse effects across all four groups.

## Discussion

Male infertility has emerged as a notable concern in healthcare, and research indicates that its occurrence is on the rise. The problem is influenced by both genetic and environmental elements, although the specific cause remains unclear in approximately 40-50% of cases. Scientific findings suggest that the quantity and quality of spermatogenesis play a vital role in the development of male infertility. A good prognosis in this regard has been associated with increased sperm motility and count, and improved sperm morphology (Ayaz et al., 2018; Skakkebaek et al., 2016; Nasimi Doost Azgomi et al., 2018).

To the best of our knowledge, this study represents the first research to compare the effects of carrot, and clomiphene on sperm parameters in cases of idiopathic male infertility. In this particular study, the administration of carrot seeds for a duration of 90 days resulted in improved sperm parameters among men experiencing infertility. The findings of the study demonstrated significantly improvements in total sperm count, sperm concentration, total motility, total motile sperm count (TMSC) (Clomiphene/Carrot, and Clomiphene/Carrot/Lifestyle modification groups), and progressive motility, normal morphology (Clomiphene/Carrot/ modification group). Our findings showed a significant difference in some sperm parameters between patients in the clomiphene + carrot + lifestyle modification group and the clomiphene group (as a control group). We found that simultaneous use of carrot and lifestyle modification significantly increased the mean total sperm count, sperm concentration and TMSC compared to the control group.

Consistent with these findings, the previous research indicated that different components of the carrot plant, particularly its seeds, have the potential to improve sperm count and motility in infertile males (Ouladsahebmadarek et al., 2016). Many studies have been conducted on the chemical composition of carrot plant, revealing the presence of numerous active ingredients such as carotenoids, flavonoids, tannins, volatile oils, and steroids (Ahmad et al., 2019; Ganguly et al., 2020). Studies have reported various effects of carrots, including their ability to enhance male potency and stimulate menstruation in women (MousaviKhorshidi et al., 2023). Furthermore, it has been observed that the seeds of the plant exhibit greater effectiveness compared to other parts (Nouri et al., 2009).

Carrots have also been investigated for their potential to improve female sexual dysfunction. In a particular research, participants in the study group were administered with 500 mg of carrot seeds three times a day for duration of 12 weeks, while a control group received a placebo. The effectiveness of the treatments was evaluated by comparing the results of completed female sexual function index questionnaires between the two groups. The described treatment showed no reported side effects, which could be seen as an advantage compared to pharmaceutical interventions (Sadeghi et al., 2020). The extract derived from carrot seeds is known to possess higher levels of antioxidants compared to other parts of plant and has the potential to increase sperm reserves in the cauda epididymis (CESR) (Yu et al., 2005). Moreover, studies have indicated that co-administration of this extract with gentamicin can help counteract the negative effect of gentamicin on sperm count (Nouri et al., 2009; Yu et al., 2005).

Exposure to carrot seed extract has been shown to enhance spermatogenic activity and increase the number of developing germ cells, suggesting a positive effect on the process of meiosis. Sperm quality, particularly normal sperm morphology, plays a crucial role in successful fertilization of the oocyte, leading to higher pregnancy rates and a reduced risk of fetal DNA damage. Furthermore, the improvement in sperm quality holds significant prognostic value in assisted reproduction techniques and provides notable benefits for *in vitro* fertilization (IVF) patients who have limited sperm quality, especially in terms of sperm morphology (Agarwal et al., 2014; Aitken et al., 2014; Kolangi et al., 2019).

Several studies have indicated that the administration of carrot seed extract can lead to an increase in testosterone levels, suggesting its potential to enhance spermatogenesis hormones (Nouri et al., 2009). This indicates that the use of carrot seed extract may be an effective and safe approach to mitigate the toxic effects of chemicals on the reproductive system and male infertility. By utilizing this plant, it is possible to potentially alleviate the negative impact of harmful substances on male fertility (Nouri et al., 2009; Mohammadi et al., 2013). In a study conducted by Kosuri et al., the antioxidant activity of various parts of the *D. carota* plant was investigated. The essential oil of the seed was found to contain cedarone S (14.04%), geranyl acetate (52.45%), and azarone E (11.39%) as its main components. Additionally, the seed extract of *D. carota* was found to contain several phenolic compounds, including gallic acid. They observed significant antioxidant activity in both the essential oil and the methanolic extract of the *D. carota* seed, with the methanolic extract demonstrating a more pronounced antioxidant effect (Ksouri et al., 2015).

Clomiphene citrate is a widely used drug in the field of fertility (Rizzuti et al., 2023). It acts as an estrogen agonist, meaning it competes with estradiol (E2) for estrogen receptors in the hypothalamus. By doing so, it blocks the negative feedback effect of circulating E2 on the hypothalamic-pituitary-testicular (HPT) axis. As a result, clomiphene increases luteinizing hormone (LH) levels, stimulates the production of endogenous testosterone (T), and promotes spermatogenesis (Puia and Pricop, 2022). The dosing regimen for clomiphene can vary, typically ranging from 12.5 to 50 mg/d. However, most studies administered doses between 12 and 25 mg/d (Sharma et al., 2019). It has been observed that doses higher than 50 mg/d do not necessarily result in a better response, because the agonistic effect of zuclomiphene, a metabolite of clomiphene citrate, can diminish the antagonist effect on estrogen receptors responsible for increasing positive feedback on the HPT axis (Fontenot et al., 2016).

In this study, the patient took a daily dose of 50 mg of clomiphene for a duration of 90 days. Generally, this dose was well tolerated by the patient, who did not report any visual symptoms or immediate abdominal discomfort. However, it is worth noting that the benefits of clomiphene are not universally guaranteed, as impaired semen analysis can still be observed in certain individuals who undergo clomiphene treatment. According to a systematic review that included 384 subfertile men from 11 cohorts, it was reported that 19% of clomiphene-treated subjects experienced a decrease in sperm count, 21% experienced a decrease in sperm concentration, 17% experienced a decrease in sperm motility, and 24% experienced a decrease in total motile sperm count (Gundewar et al., 2021). These findings highlight that while clomiphene can be effective for some individuals, it may not lead to improvements in sperm parameters for all patients and could potentially result in a decline in certain aspects of sperm quality. Additionally, it has been observed that after discontinuing clomiphene treatment, semen parameters did not recover in up to 17% of subjects, indicating a potential long-lasting impact on fertility. Nevertheless, the side effects of clomiphene are generally considered mild and well tolerated. The most common side effects reported include headaches, blurred vision, dizziness, vomiting, nausea, gynecomastia (enlargement of breast tissue in males), weight gain, hypertension (high blood pressure), and in some cases, a paradoxical decrease in total testosterone levels. It is important to note that the occurrence and severity of side effects can vary among individuals (Gundewar et al., 2021; Rizzuti et al., 2023).

The educational program in the study included various lifestyle modifications such as time of intercourse, preparation of the wife before intercourse, having sex only when there is a real desire to have intercourse, appropriate frequency of intercourse, compliance with the necessary conditions after intercourse, enhancement of potency and adjusting sleep and following a daily exercise pattern. The study results indicated that the combination of carrot supplementation and lifestyle modifications had more pronounced effects on sperm parameters compared to lifestyle modifications alone. This suggests that the inclusion of carrot supplementation in addition to lifestyle changes yielded significant effects in sperm parameters. In the context of idiopathic male infertility, the application of traditional Persian medicine-based education resulted in improvements in various domains of sperm parameters, with the exception of semen volume. It is worth noting that there is limited research available on the effects of healthy lifestyle education specifically based on temperament programs and interventions, including nutrition counseling approaches, on sexual dysfunction (Karimi‑valoujaei et al., 2022). Therefore, further studies are needed to explore the impact of these educational programs on sexual dysfunction and related outcomes. Indeed, the World Health Organization (WHO) recognizes the importance of traditional medicine and encourages its development and integration into healthcare systems (Kasilo and Nikiema, 2014). Traditional medicine, including practices rooted in local cultures and traditions, is often well-received by the public and has been found to provide safe and cost-effective healthcare services (Rezaeizadeh et al., 2009; Ebrahimiam et al. 2023). By acknowledging the value of traditional medicine and promoting its utilization, the WHO aims to enhance access to comprehensive healthcare options and improve overall health outcomes for individuals and communities.

In this study, no side effects were reported in relation to the consumption of carrot seeds. This finding suggests that carrot seed consumption may have an advantage over conventional pharmacological interventions for idiopathic male infertility, as it appears to be well-tolerated and devoid of adverse effects. However, it is essential to note that this conclusion is specific to the study mentioned, and further research and clinical trials are necessary to validate the safety and efficacy of carrot seed consumption in the context of male infertility.

There were several limitations in this study. Initially, we faced the constraint of not being able to conduct long-term patient follow-ups. Additionally, the administration method utilized in this study involved using capsules to deliver Carrot seeds to the patients, which diverges from the traditional approach employed by Persian practitioners when administering medication. It is plausible that this disparity could have influenced the study's outcome.

The findings of this study suggest that the consumption of capsules containing carrot extract, either alone or in combination with lifestyle education may potentially enhance sperm parameters in cases of idiopathic infertility among men. Given the widespread societal acceptance of carrot, this treatment option holds promise as a cost-effective alternative for addressing idiopathic male infertility. However, in order to establish a more comprehensive understanding of the clinical implications of these findings, further research with larger sample sizes and longer durations is necessary. Future studies may also benefit from investigating fertilization and pregnancy rates, exploring the molecular mechanisms of this plant, examining its bioactive constituents, and conducting animal studies.

**Table 1:** Microbial tests of carrot seed extract

**Test**	**Specification**	**Result**
**Total aerobic microbial count**	Dose not exceed 10^5^ cfu/g	1.5*10
**Total combined molds and yeasts count**	Dose not exceed 10^4^ cfu/g	<10
**Bile-tolerant gram-negative bacteria**	Dose not exceed 10^4^ cfu/g	<10
**Escherichia coli**	Absence	Conform
**Salmonella species**	Absence	Conform

**Table 2:** Baseline demographic characteristics of study groups

**Characteristics**		**Clomiphene**	**Clomiphene+Carrot**	**Clomiphene+Lifestyle**	**Clomiphene+** **Carrot+Lifestyle**	**p-value**
BMI, kg/m2 (Mean ± SD)		26.5±3.7	25.7±3.7	26.2±4.0	24.3±5.4	0.49*
Education status, no. (%)						
	Less than high school	4 (26.7%)	4 (26.7%)	4 (26.7%)	7 (46.7%)	0.51**
	High school diploma	5 (33.3%)	4 (26.7%)	8 (53.3%)	5 (33.3%)	
	Bachelor’s degree or higher	6 (40.0%)	7 (46.7%)	3 (20.0%)	3 (20.0%)	
Place of residence, no. (%)						
	Urban	13 (86.7%)	14 (93.3%)	14 (93.3%)	14 (93.3%)	0.88***
	Rural	2 (13.3%)	1 (6.7%)	1 (6.7%)	1 (6.7%)	
Employment, no. (%)						
	Employee	4 (26.7%)	4 (26.7%)	2 (13.3%)	2 (13.3%)	0.83**
	Worker	3 (20.0%)	1 (6.7%)	3 (20.0%)	3 (20.0%)	
	Self-employment	8 (53.3%)	10 (66.7%)	10 (66.7%)	10 (66.7%)	

* ANOVA ** Chi-Square *** Fisher's exact test

**Table 3:** Intragroup comparison of sperm parameters before and after intervention in the studied groups

**p-value**	**Clomiphene+** **Carrot+Lifestyle**	**Clomiphene+Lifestyle**	**Clomiphene+Carrot**	**Clomiphene**		**Variables**
0.071^c^	3.35±1.43	3.01±1.01	2.37±1.21	2.63±1.07	Pre‐intervention	**Semen volume (ml)**
0.765^e^	3.34±1.50	2.94±1.55	2.47±1.16	2.55±1.30	Post‐intervention	
	0.98^a^	0.82^a^	0.76^a^	0.80^a^	p value	
0.354^d^	71.24±99.9	151.77±225.65	149.30±229.28	119.36±122.91	Pre‐intervention	**Total sperm count (×10** ^6^ **)**
0.082^f^	132.75±146.21	164.37±255.32	255.26±255.50	78.71±52.86	Post‐intervention	
	0.01^a^	0.97^b^	0.01^a^	0.21^b^	p value	
0.295^d^	28.80±52.04	68.59±107.15	44.84±53.49	40.41±35.84	Pre‐intervention	**Sperm concentration (×10** ^6^ **)**
0.007^f^	58.81±73.70	51.83±71.12	83.02±77.12	32.67±23.44	Post‐intervention	
	0.001^a^	0.95^b^	0.03^a^	0.82^b^	p value	
0.812^c^	22.14±13.20	26.34±13.76	23.88±19.65	27.02±14.28	Pre‐intervention	**Progressive motility (%)**
0.868^e^	32.83±16.08	19.89±14.92	31.77±18.97	24.81±15.28	Post‐intervention	
	0.002^a^	0.21^a^	0.13^a^	0.60^a^	p value	
0.833^c^	41.09±21.54	45.62±20.38	42.72±20.84	45.62±18.08	Pre‐intervention	**Total Sperm motility (%)**
0.805^e^	59.97±23.42	37.69±28.83	54.96±24.63	43.53±22.25	Post‐intervention	
	0.001^a^	0.21^a^	0.02^a^	0.67^a^	p value	
0.748^d^	32.33±52.04	84.63±144.08	74.29±127.84	62.68±83.09	Pre‐intervention	**TMSC (×10** ^6^ **)**
0.021^f^	101.59±100.59	97.19±191.76	139.62±179.21	32.70±33.33	Post‐intervention	
	0.002^b^	0.68^b^	0.003^a^	0.53^b^	p value	
0.759^d^	8.92±10.67	9.99±16.96	10.13±10.32	9.31±17.83	Pre‐intervention	**Normal morphology (%)**
0.541^f^	12.01±11.93	11.49±18.83	11.79±9.40	7.04±9.87	Post‐intervention	
	0.01^b^	0.28^b^	0.07^b^	0.50^b^	p value	

TMSC: Total Motility Sperm Count; a: Paired t-test ;b: Wilcoxon signed-rank test; c: ANOVA ; d: Kruskal-Wallis ; e:ANCOVA; f: GEE

**Table 4:** Comparison of sperm parameters after intervention between clomiphene group and intervention groups

**Variables**	**Clomiphene+Carrot** **(p-Value)**	**Clomiphene+Lifestyle** **(p-Value)**	**Clomiphene+Carrot+Lifestyle** **(p-Value)**
Semen volume (mL)	0.97	0.79	0.55
Total sperm count (×10^6^)	0.19	0.81	0.01
Sperm concentration (×10^6^)	0.12	0.36	0.007
Progressive motility (%)	0.77	0.99	0.25
Total Sperm motility (%)	0.51	0.99	0.08
TMSC (×10^6^)	0.07	0.72	0.002
Normal morphology (%)	0.68	0.77	0.43

## Ethics approval and consent to participate

The authors confirm that all experiments were performed in accordance with relevant guidelines and regulations. Also, they confirming that informed consent was obtained from all subjects. All the methods were in accordance with the declaration of Helsinki, and all methods were conducted in accordance with relevant guidelines and regulations. This randomized double-blind controlled clinical trial was approved by the Committee of Medical ethics, Golestan University of Medical Science (approval code: IR.GOUMS.REC.1400.319), and was registered by the Iranian Registry of Clinical Trials (registration ID: IRCT20210911052430N1).

## References

[1] Abubakar AR, Haque M (2020). Preparation of medicinal plants: basic extraction and fractionation procedures for experimental purposes. J Pharm Bioallied Sci.

[2] Abouzaripour M, Daneshi E, Saeid Miri S (2024). Effect of nigella sativa on dexamethasone-induced testicular toxicity in mice. Avicenna J Phytomed.

[3] Agarwal A, Tvrda E, Sharma R (2014). Relationship amongst teratozoospermia, seminal oxidative stress and male infertility. Reprod Biol Endocrinol.

[4] Ahmad MK, Mahdi AA, Shukla KK, Islam N, Rajender S, Madhukar D, Shankhwar SN, Ahmad S (2010). Withania somnifera improves semen quality by regulating reproductive hormone levels and oxidative stress in seminal plasma of infertile males. Fertil Steril.

[5] Ahmad T, Cawood M, Iqbal Q, Ariño A, Batool A, Tariq RMS, Azam M, Akhtar S (2019). Phytochemicals in daucus carota and their health benefits. Foods.

[6] Ahmadi-Asrbadr Y, Hemmati-Ghavshough M, Khanzadeh N, Ansari F, Mohammad-Rahimi M (2022). Comparison of the effect of combined therapy of hcg ampule and letrozole tablet with each method separately on the spermiogram parameters in the obese men with idiopathic infertility: a clinical trial. Am J Clin Exp Urol.

[7] Aitken R, Finnie J, Muscio L, Whiting S, Connaughton H, Kuczera L, Rothkirch T, De iuliis G (2014). Potential importance of transition metals in the induction of dna damage by sperm preparation media. Hum Reprod.

[8] Alahmar AT (2018). The effects of oral antioxidants on the semen of men with idiopathic oligoasthenoteratozoospermia. Clin Exp Reprod Med.

[9] Alfaro gómez M, Fernández-Santos MDR, Jurado-Campos A, Soria-Meneses PJ, Montoro angulo V, Soler AJ, Garde JJ, Rodríguez-Robledo V (2023). On males, antioxidants and infertility (moxi): certitudes, uncertainties and trends. Antioxidants (Basel).

[10] Alshahrani S, Aldossari K, Al‐Zahrani J, Gabr A, Henkel R, Ahmad G (2018). Interpretation of semen analysis using who 1999 and who 2010 reference values: abnormal becoming normal. Andrologia.

[11] Aqili khorasani SMHIMH (1385). Kholasatol hekmat.

[12] Arzani M (1391). Mofarreh- al gholub 2.

[13] Arzani MA (2008). Tebbe akbari 2.

[14] Ayaz A, Kothandaraman N, Henkel R, Sikka SC (2018). Impact of environmental factors on the genomics and proteomics landscapes of male infertility. Bioenvironmental issues affecting men's reproductive and sexual health.

[15] Bjorndahl L, Brown JK (2022). The sixth edition of the who laboratory manual for the examination and processing of human semen: ensuring quality and standardization in basic examination of human ejaculates. Fertil Steril.

[16] Campbell MF, Walsh PC, Wein AJ, Partin AW, Dmochowski R, Kavoussi LR, Peters CCA (2021). Campbell-walsh-wein urology.

[17] Cooper TG, Noonan E, Von Eckardstein S, Auger J, Baker H, Behre HM, Haugen TB, Kruger T, Wang C, Mbizvo MT, Vogelsong KM (2010). World health organization reference values for human semen characteristics. Hum Reprod Update.

[18] Ebrahimian A, Rahbar S, Homami S, Paknazar F, Fakhr-Movahedi A (2023). Comparison of the effect of mint extract and chamomile drops on the gastric residual volume of traumatic patients under mechanical ventilation and nasogastric tube feeding in the intensive care unit: a triple-blind, randomized crossover trial. Avicenna J Phytomed.

[19] Fontenot GK, Wiehle RD, Podolski JS (2016). Differential effects of isomers of clomiphene citrate on reproductive tissues in male mice. BJU Int.

[20] Ganguly M, Hazarika J, Sarma S, Bhuyan P, Mahanta R (2020). Estrogen receptor modulation of some polyphenols extracted from daucus carota as a probable mechanism for antifertility effect: an in silico study. J Theor Comput Chem.

[21] Godmann M, Lambrot R, Kimmins S (2009). The dynamic epigenetic program in male germ cells: Its role in spermatogenesis, testis cancer, and its response to the environment. Microsc Res Tech.

[22] Gundewar T, Kuchakulla M, Ramasamy R (2021). A paradoxical decline in semen parameters in men treated with clomiphene citrate: A systematic review. Andrologia.

[23] Guo DP, Zlatev DV, Li S, Baker LC, Eisenberg ML (2020). Demographics, Usage Patterns, and Safety of Male Users of Clomiphene in the United States. World J Mens Health.

[24] Gupta A, Mahdi AA, Shukla KK, Ahmad MK, Bansal N, Sankhwar P, Sankhwar SN (2013). Efficacy of withania somnifera on seminal plasma metabolites of infertile males: A proton nmr study at 800 mhz. J Ethnopharmacol.

[25] Huijben M, Huijsmans RL, Lock MTW, De kemp VF, De kort LM, Van breda JH (2023a). Clomiphene citrate for male infertility: a systematic review and meta‐analysis. Andrology.

[26] Huijben M, Lock M, De kemp V, Beck J, De kort L, Van breda H (2023b). Clomiphene citrate: A potential alternative for testosterone therapy in hypogonadal males. Endocrinol Diabetes Metab.

[29] Karimi‑Valoujaei S, Kashi Z, Yousefi SS, Nia HS, Khani S (2022). The effect of a education-counseling program based on temperament in iranian traditional medicine on sexual dysfunction in diabetic women. J Nurs Midwifery Sci.

[30] Kolangi F, MousaviKhorshidi A (2023). Daucus carota : Can it be offered as an alternative treatment for improving semen quality. JCBR.

[31] Kolangi F, Shafi H, Memariani Z, Kamalinejad M, Bioos S, Jorsaraei SGA, Bijani A, Shirafkan H, Mozaffarpur SA (2019). Effect of alpinia officinarum hance rhizome extract on spermiogram factors in men with idiopathic infertility: A prospective double‐blinded randomised clinical trial. Andrologia.

[32] Ksouri A, Dob T, Belkebir A, Krimat S, Chelghoum C (2015). Chemical composition and antioxidant activity of the essential oil and the methanol extract of algerian wild carrot daucus carota l ssp carota (l) thell. J Mater Environ Sci.

[33] Lindsay TJ, Vitrikas KR (2015). Evaluation and treatment of infertility. Am Fam Physician.

[34] Maohamaad S (1388). Lezzat-al-vesal.

[35] Mohammadi F, Nikzad H, Taherian A, Amini mahabadi J, Salehi M (2013). Effects of herbal medicine on male infertility. Anat Sci J.

[36] MousaviKhorshidi A, Enayati A, Kolangi F (2023). A comprehensive review of scientific evidence of Daucus carota L plant from the viewpoints of Persian Medicine and Current Medicine: A review study. jiitm.

[37] Nasimi doost azgom R, Nazemiyeh H, Sadeghi Bazargani H, Fazljou S, Nejatbakhsh F, Moini Jazani A, Ahmadi asrbadr Y, Zomorrodi A (2018). Comparative evaluation of the effects of withania somnifera with pentoxifylline on the sperm parameters in idiopathic male infertility: a triple‐blind randomised clinical trial. Andrologia.

[38] Nejatbakhsh F, Shirbeigi L, Rahimi R, Abolhassani H (2016). Review of local herbal compounds found in the Iranian traditional medicine known to optimise male fertility. Andrologia.

[39] Nouri M, Khaki A, Fathiazar F, Rashidi MR (2009). The protective effects of carrot seed extract on spermatogenesis and cauda epididymal sperm reserves in gentamicin treated rats. Yakhteh Med J.

[40] Oliveira KG, Miranda SA, Leão DL, Brito AB, Santos RR, Domingues SF (2011). Semen coagulum liquefaction, sperm activation and cryopreservation of capuchin monkey (cebus apella) semen in coconut water solution (cws) and tes–tris. Anim Reprod Sci.

[41] Ouladsahebmadrek E, Seyed Ghiasi G, Khaki A, Ahmadi Y, Farzad L, Ghasemzadeh A, Hajizade K (2016). The effect of compound herbal remedy used in male infertility on spermatogenesis and pregnancy rate. Int J Women's Health Reprod Sci.

[42] Panner selvam MK, Baskaran S, Tannenbaum Greenberg J, Shalaby HY, Hellstrom WJ, Sikka C (2023). Clomiphene citrate in the management of infertility in oligospermic obese men with hypogonadism: retrospective pilot study. Medicina.

[43] Puia D, Pricop C (2022). Effectiveness of clomiphene citrate for improving sperm concentration: a literature review and meta-analysis. Cureus.

[44] Rezaeizadeh H, Alizadeh M, Naseri M, Shams AM (2009). The traditional Iranian medicine point of view on health and disease. Iran J Public Health.

[45] Rizzuti A, Alvarenga C, Stocker G, Fraga L, Santos HO (2023). Early pharmacologic approaches to avert anabolic steroid-induced male infertility: a narrative review. Clin Ther.

[46] Sadeghi S, Bahrami R, Raisi F, Rampisheh Z, Ghobadi A, Akhtari E (2020). Evaluation of the effect of carrot seed (daucus carota) in women of fertile age with hypoactive sexual desire disorder: a randomized double-blind clinical trial. Complement Ther Med.

[47] Sharma D, Zillioux J, Khourdaji I, Reine K, Wheeler K, Costabile R, Kavoussi P, Smith R (2019). Improvements in semen parameters in men treated with clomiphene citrate-a retrospective analysis. Andrologia.

[49] Skakkebaek NE, Rajpert-De meyts E, Buck louis GM, Toppari J, Andersson AM, Eisenberg ML, Jensen TK, Jørgensen N, Swan SH, Sapra KJ (2016). Male reproductive disorders and fertility trends: influences of environment and genetic susceptibility. Physiol Rev.

[50] Surampudi P, Swerdloff RS, Wang C (2014). An update on male hypogonadism therapy. Expert Opin Pharmacother.

[51] Tahvilzadeh M, Hajimahmoodi M, Toliyat T, Karimi M, Rahimi R (2016). An evidence‐based approach to medicinal plants for the treatment of sperm abnormalities in traditional p ersian medicine. Andrologia.

[52] Thaker H, Ko EY, Sabanegh ES, Brannigan RE, Alukal JP, Samplaski MK (2020). Empirical medical therapy for idiopathic male infertility. F S Rep.

[54] Tweij TAR (2015). Comparison between the effect of clomid and pregnyl on sperm concentration, percentage of sperm progressive and percentage of normal sperm morphology. Adv Life Sci Technol.

[55] Yu LL, Zhou KK, Parry J (2005). Antioxidant properties of cold-pressed black caraway, carrot, cranberry, and hemp seed oils. Food chem.

